# Long-term performance monitoring of a secondary electrospray ionisation high-resolution mass spectrometry platform using a vapor generator for routine quality control

**DOI:** 10.1039/d6ay01843b

**Published:** 2026-10-09

**Authors:** Albin Vadakkechira, Renato Zenobi, Daniel Pohl, Kathryn J. Burton-Pimentel, Stamatios Giannoukos

**Affiliations:** a Department of Chemistry and Applied Biosciences, ETH Zürich Vladimir-Prelog-Weg 3 Zurich 8093 Switzerland stamatios.giannoukos@org.chem.ethz.ch +41 44 632 61 12; b Department of Gastroenterology and Hepatology, USZ Zurich Switzerland; c Agroscope Bern Switzerland

## Abstract

Secondary electrospray ionisation high-resolution mass spectrometry (SESI-HRMS) is a powerful, non-invasive tool for real-time breath analysis. However, instrumental drift, capillary degradation and other technical variations compromise clinical applications, especially for long-term studies, which require robust quality control (QC) methodologies. In this work, we present the use of an evaporation chamber-based reference gas system to monitor SESI-MS system performance over almost two years. Targeted QC measurements with deuterated butyric acid were recorded three times on each measurement day. Analytical stability was systematically quantified across both positive and negative ion modes. In a standalone continuous characterization, the reference gas generator delivered a stable signal over several hours. A Linear Mixed-Effects Model (LMM) was constructed to evaluate the influence of time-of-day on instrument performance. This revealed significant effects: positive mode signal intensity progressively increased throughout the day, whereas for negative mode, an initial drop in signal intensity and subsequent stabilisation was observed. Furthermore, the longitudinal QC framework successfully tracked capillary performance over time, providing objective performance metrics to retrospectively evaluate the impact of targeted hardware maintenance on system performance. The implementation of a structured QC protocol provides a valid methodology for long-term SESI-HRMS performance monitoring, ensuring data integrity and supporting the translation of SESI-HRMS into large-scale clinical applications.

## Abstract 2

Secondary electrospray ionisation high-resolution mass spectrometry (SESI-HRMS) is a powerful, non-invasive tool for real-time breath analysis.

## Graphical abstract

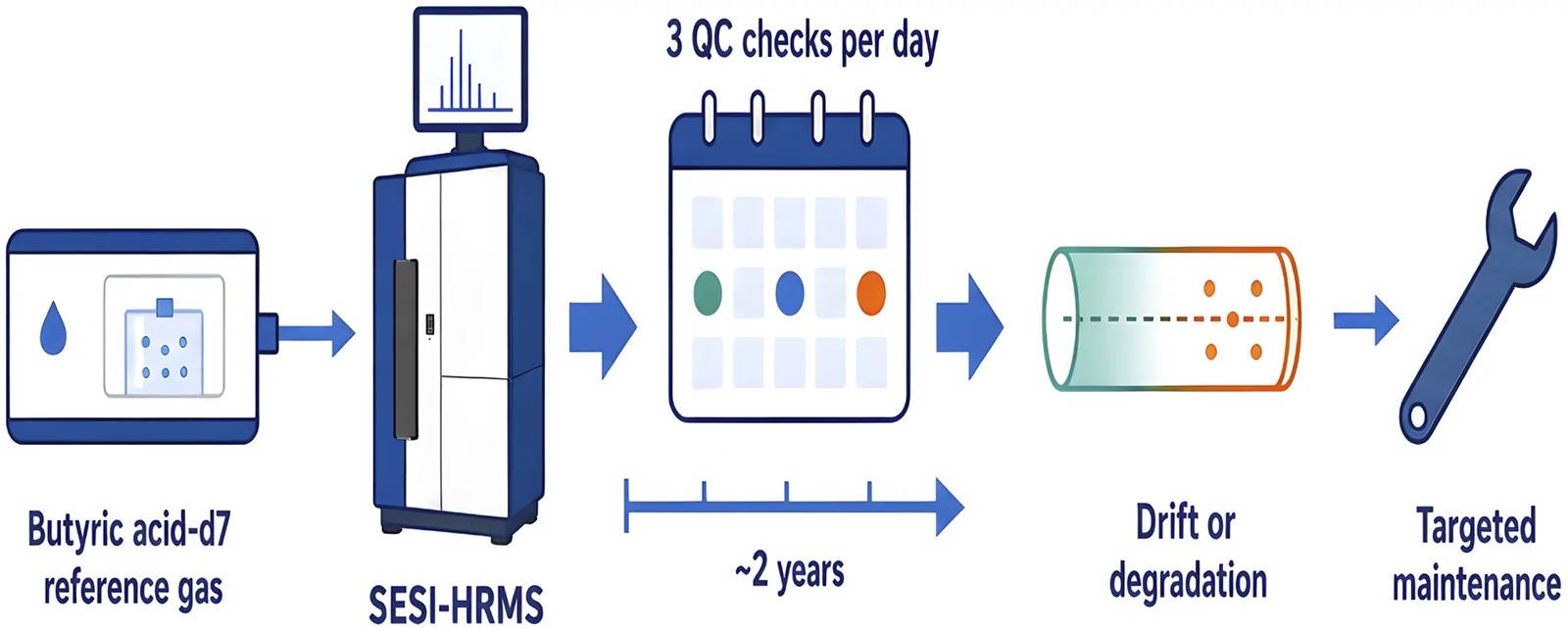

## 1 Introduction

Breath analysis has considerable potential for non-invasive clinical diagnostics, therapeutic monitoring, and biomarker discovery, as exhaled breath contains rich, dynamic chemical information that reflects endogenous metabolic processes, host–microbiome interactions, environmental exposures, and disease-related biochemical processes.^1^ Secondary electrospray ionisation coupled to high-resolution mass spectrometry (SESI-HRMS) has emerged as a highly promising analytical platform for real-time breath analysis, offering direct, sensitive, and broad detection of volatile and semi-volatile metabolites without the need for sample pre-concentration or extensive sample preparation.^2,3^ These characteristics make SESI-HRMS highly attractive for longitudinal clinical studies, where repeated breath measurements can provide time-resolved metabolic information under minimally invasive conditions.

However, the translation of SESI-HRMS into large-scale clinical studies and, ultimately, routine clinical practice is currently compromised by challenges related to analytical robustness, long-term stability, and standardization of sampling procedures.^4^ Unlike conventional offline workflows, online SESI-HRMS measurements rely on the continuous, reproducible operation of multiple interconnected components, including the electrospray source, transfer line, ionization chamber, gas flow control, and high-resolution mass spectrometer. Over extended periods of time, technical factors such as electrospray emitter aging or replacement, source contamination, changes in ion transmission, system drift, environmental fluctuations, and routine maintenance interventions can introduce substantial analytical variability. Such variability may obscure biological effects, introduce batch effects, or lead to incorrect biomarker interpretation if not adequately monitored. Robust quality control (QC) procedures are essential for ensuring data comparability across measurement days, study phases, and clinical cohorts. This is particularly important in longitudinal clinical studies conducted over several months or years, where samples are acquired under inherently changing instrumental and operational conditions, making them particularly susceptible to batch effects.^5,6^ Advanced downstream data analysis approaches rely on the assumption that measured differences primarily reflect biological variation rather than uncontrolled technical variability. Consequently, longitudinal QC strategies are required not only to monitor instrument performance but also to identify analytical shifts, support batch-effect correction, and provide objective criteria for evaluating data integrity.

Reference gas standards represent a promising approach for routine QC in real-time breath analysis, as they allow repeated assessment of signal intensity, mass accuracy, temporal stability, and system responsiveness under controlled conditions. In contrast to biological QC samples, reference gas standards can be generated reproducibly, measured frequently, and linked directly to instrumental performance.^7–9^ Such standards are particularly valuable for SESI-HRMS, where the ionisation efficiency and signal response may be affected by humidity, gas flows, source conditions, and electrospray performance. However, despite the increasing use of SESI-HRMS in clinical and metabolic studies, systematic evaluations of long-term QC strategies remain scarce. Previous work by Singh *et al.*^10^ has provided important insights into SESI-HRMS stability, including short-term technical variability using a reactive gas standard generator and β-pinene as a reference substance, measuring continuously for one h. Additionally, they investigated the reproducibility of breath measurements over approximately one month using three classes of aldehydes (4-hydroxy-2-alkenals, 2-alkenals and 4-hydroxy-2,6-alkedienals) in exhaled breath, showing that inter-individual differences dominate variability in the data, rather than intra-subject variability. However, there remains a lack of evidence regarding how SESI-HRMS platforms behave over extended clinical study periods, in which performance must be evaluated across short-term, daily, and long-term timescales to distinguish acceptable instrumental variation from systematic drift or abrupt changes caused by specific maintenance events or instrument interventions.

The present study aims to evaluate the long-term performance of a SESI-MS platform during the Lactobreath clinical study^11^ using a custom-built reference gas generation system^12^ implemented as a routine QC strategy over a 21 month period encompassing 112 measurement days. Deuterated butyric acid was used as the reference substance to monitor the analytical performance throughout the study. The aims of this study are threefold: (i) to assess short-term, daily, and long-term instrument performance, (ii) to evaluate potential relationships between observed analytical changes and documented instrument interventions and maintenance history, and (iii) to evaluate the suitability and reliability of the reference gas generation system as a longitudinal QC strategy. By providing a detailed longitudinal assessment of SESI-MS performance, this study contributes to the development of robust QC practices and frameworks required for reliable breath metabolomics in long-term clinical studies.

## 2 Experimental section

### 2.1 Motivation and clinical study context

The present work was conducted in the context of the Lactobreath study, a pilot clinical study designed to identify exhaled breath biomarkers associated with lactose malabsorption and lactose intolerance.^11^ Because breath measurements were acquired over a period of 21 months, a robust external QC strategy was required to monitor SESI-MS performance and ensure comparability of data collected across the entire study period. We implemented a protocol to repeatedly measure a reference compound on each clinical measurement day as an external QC marker of instrument performance. This approach enabled the temporal evolution of the analytical response to be assessed across hourly, daily (between-day), and long-term (monthly to annual) timescales. Additionally, to evaluate the stability of the reference gas system itself, a prolonged continuous measurement of 3 h was performed for both ionisation modes after completion of the study. In addition, a 24 h continuous measurement in positive ion mode was performed before the start of the study. These continuous experiments characterise the reference gas generator independently and are not intended to validate the QC measurements acquired during the clinical study. Deuterated butyric acid was selected as the reference compound as butyric acid is a volatile organic compound previously reported in exhaled breath,^13^ while its deuterated analogue provides a distinguishable signal that can be monitored independently from endogenous breath-derived features. The repeated measurement of a defined reference gas therefore served as a proxy for technical variation in the SESI-MS platform under conditions relevant to breath analysis.

### 2.2 Reagents

All aqueous solutions were prepared using Optima LC-MS grade water (Fisher Scientific, USA). The electrospray solution was prepared by dissolving formic acid (purity ≥ 99.99%, Sigma-Aldrich, USA) in water to a concentration of 0.1% v/v. D_7_-Butyric acid (purity ≥ 98%, Cambridge Isotope Laboratories, USA) at a volume concentration of 0.2% v/v was prepared and used to generate the gas standard. Equipment was cleaned and rinsed with a 1 : 1 (v/v) mixture of Optima LC-MS grade water and methanol (Fisher Scientific, USA).

### 2.3 Experimental setup

The custom-built gas standard addition system and its application used in this study have been described previously.^12^ Briefly, the system was used to generate a humidified nitrogen flow containing a defined D_7_-butyric acid gas standard under conditions designed to mimic human exhaled breath. The bubbler used to humidify the in-house nitrogen supply was maintained at 37 °C, while the stainless-steel enclosure containing the tubing was heated to 60 °C. The outlet of the system was connected to an EXHALION flow meter (Fossil Ion Tech, Spain) and a SuperSESI ion source (Fossil Ion Tech, Spain), which was mounted on a Q-Exactive Plus Orbitrap mass spectrometer (Thermo Fisher, Germany).

The SESI ion source sampling line and ionization chamber were heated to 130 °C and 90 °C, respectively. The electrospray was generated from an aqueous formic acid solution (0.1% v/v), using a nano-electrospray capillary with an internal diameter of 20 µm (Fossil Ion Tech, Spain). The spray solution was delivered using a constant pressure of 0.8 bar. The sheath gas flow was set to 15 a.u. (arbitrary units), the auxiliary gas flow was set to 2 a.u., and an electrospray voltage of ±3.5 kV was applied for positive and negative ionisation modes, respectively. The ion transfer capillary temperature was set to 250 °C, and the S-lens RF amplitude was set to 50. The automatic gain control target for the Orbitrap was set to 10^6^, and spectra were acquired at a resolution of 140 000 with a maximum injection time of 500 ms. The instrument was calibrated according to the manufacturer's operating manual, and calibration was always performed within 7 days of measurement, in accordance with vendor instructions. To ensure compatibility with the clinical breath-analysis workflow, a previously described spectral stitching^14^ acquisition approach was employed for data acquisition. Acquisition cycled through 5 scan ranges: 50–500 *m*/*z*, 50–135 *m*/*z*, 135–195 *m*/*z*, 195–277 *m*/*z*, 277–500 *m*/*z* for positive ion mode and 50–500 *m*/*z*, 50–90 *m*/*z*, 90–161 *m*/*z*, 161–255 *m*/*z*, and 255–500 *m*/*z* for negative ion mode.

External QC measurements were performed on all clinical intervention days, resulting in a total of 112 measurement days over approximately 21 months. On each day, three distinct QC measurement periods were assessed: one in the morning before clinical breath measurements began, one at the midpoint of the intervention day, and one at the end of the intervention day. This design allowed instrument performance to be evaluated both for individual measurement days and over the full study duration.

At the beginning of each intervention day, an evaporation chamber was loaded with 40 µL of D_7_-butyric acid reference solution and allowed to equilibrate for 10 min at room temperature prior to the first QC measurement. For each acquisition, the programmed flow sequence was initiated after the first 60 scans, which served as a pre-flow background for assessing carryover. This sequence consisted of 1 min of 8 L min^−1^ humidified N_2_; a combined flow of 8 L min^−1^ humidified N_2_ and 10 mL min^−1^ of D_7_-butyric acid gas standard for 10 min; and a final 1 min washout step with humidified N_2_ at 8 L min^−1^. At each QC time point, measurements were acquired first in positive-ion mode and then immediately in negative-ion mode using the same flow program. The same flow program as the external QC measurements was used to assess the system stability during a 24 h continuous monitoring experiment in positive mode before the beginning of the study and a 3 h continuous monitoring experiment for each ionisation mode, which was completed after the end of the study. In these experiments, the evaporation chamber was loaded with 50 µL of the reference standard solution and the duration of the combined humidified nitrogen and gas-standard flow was extended to 1440 min and 180 min, respectively.

Missing QC measurements (*n* = 14), when present, were documented and excluded from analyses that require complete daily triplicates. Measurements were also excluded when the extracted ion chromatogram did not follow the expected temporal profile of the programmed gas-standard introduction (*n* = 48). A detailed breakdown of the total number of planned and conducted measurements can be seen in Fig. 1. In total, 610 valid measurements (305 per ion mode) were obtained over 21 months. On 94 of the 112 intervention days, all three QC measurements could be obtained for the positive mode, while for negative mode it was possible on 87 days.

**Fig. 1:**
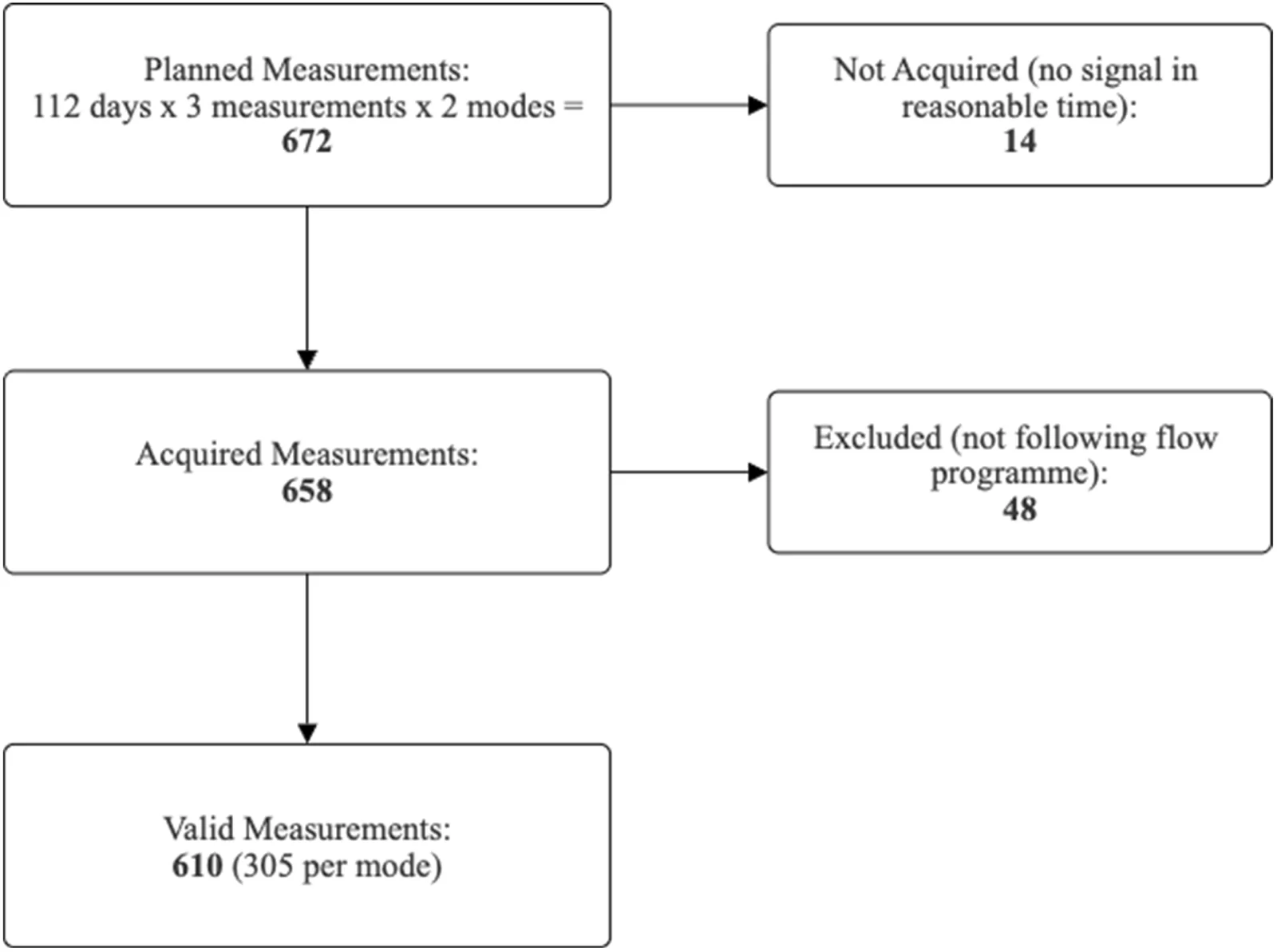
Flowchart showing the number of planned measurements, conducted measurements and missed/removed measurements.

### 2.4 Data processing and statistical analysis

The acquired RAW mass spectra files were processed in *R*,^15^ 4.6.0 using a custom script based on the *rawrr*^16^ 1.20.0 package. For each file, an extracted ion chromatogram (XIC) of either the [M + H]^+^ or [M − H]^−^ ion of D_7_-butyric acid was generated using a mass tolerance of ±7.5 ppm. XICs were visually inspected to confirm that the signal followed the expected temporal pattern corresponding to the programmed gas-standard addition and washout sequence. Measurements showing an abnormal or inconsistent signal pattern were excluded from further analysis. For each valid measurement, the signal intensity between 8 and 10 min of the acquisition was averaged using the arithmetic mean to obtain a single representative intensity value. These values were used to assess within-day variability, day-to-day variability, and long-term signal stability over the full monitoring period.

To assess operational inter- and intra-day stability, the RSD was calculated using the absolute mean intensities. For evaluation of the time-of-day effect on reference gas measurements, the intensities were log10-transformed, and a Linear Mixed-Effects Model (LMM) was employed using the *lme4* (ref. 17) 2.0.1 package in R. Daily run order was assigned as a fixed effect and treated as a categorical variable, with the specific measurement date designated as a random effect (random intercept) and using 2 degrees of freedom. Significance was determined using a global Type III ANOVA on the fitted LMM, followed by post-hoc pairwise comparisons between specific runs using the *emmeans*^18^ package (2.0.3) with *p*-values adjusted for multiple comparisons using Tukey's method. The significance threshold was *α* = 0.05. For visualisation of the data, the *ggplot2* (ref. 19) package (4.0.3) was used.

Mass accuracy was evaluated for each valid measurement as the deviation (in ppm) between the theoretical *m*/*z* of the D_7_-butyric acid [M + H]^+^ or [M − H]^−^ ion and the *m*/*z* of the most intense peak within the ±7.5 ppm extraction window, averaged over the spectra acquired between 8 and 10 min. Carryover was assessed from the signal at the target *m*/*z* during the 60 scans before introducing the reference gas in each acquisition. Because the reference gas was delivered at a single, fixed concentration and used to monitor instrument performance rather than for quantification, validation parameters of quantitative methods such as the limit of detection, limit of quantification, and calibration linearity were not determined. Selectivity was ensured by the use of a deuterated analogue that does not occur in breath or ambient air, together with high mass resolution and a narrow extraction window.

## 3. Results and discussion

### 3.1 Evaluation and utility of the reference gas system for routine QC

To quickly identify abnormal instrument performance, a Levey–Jennings chart was generated for the positive ion mode D_7_-butyric acid signal (Fig. 2). The chart for the negative mode is found in the SI (Fig. S1).

**Fig. 2:**
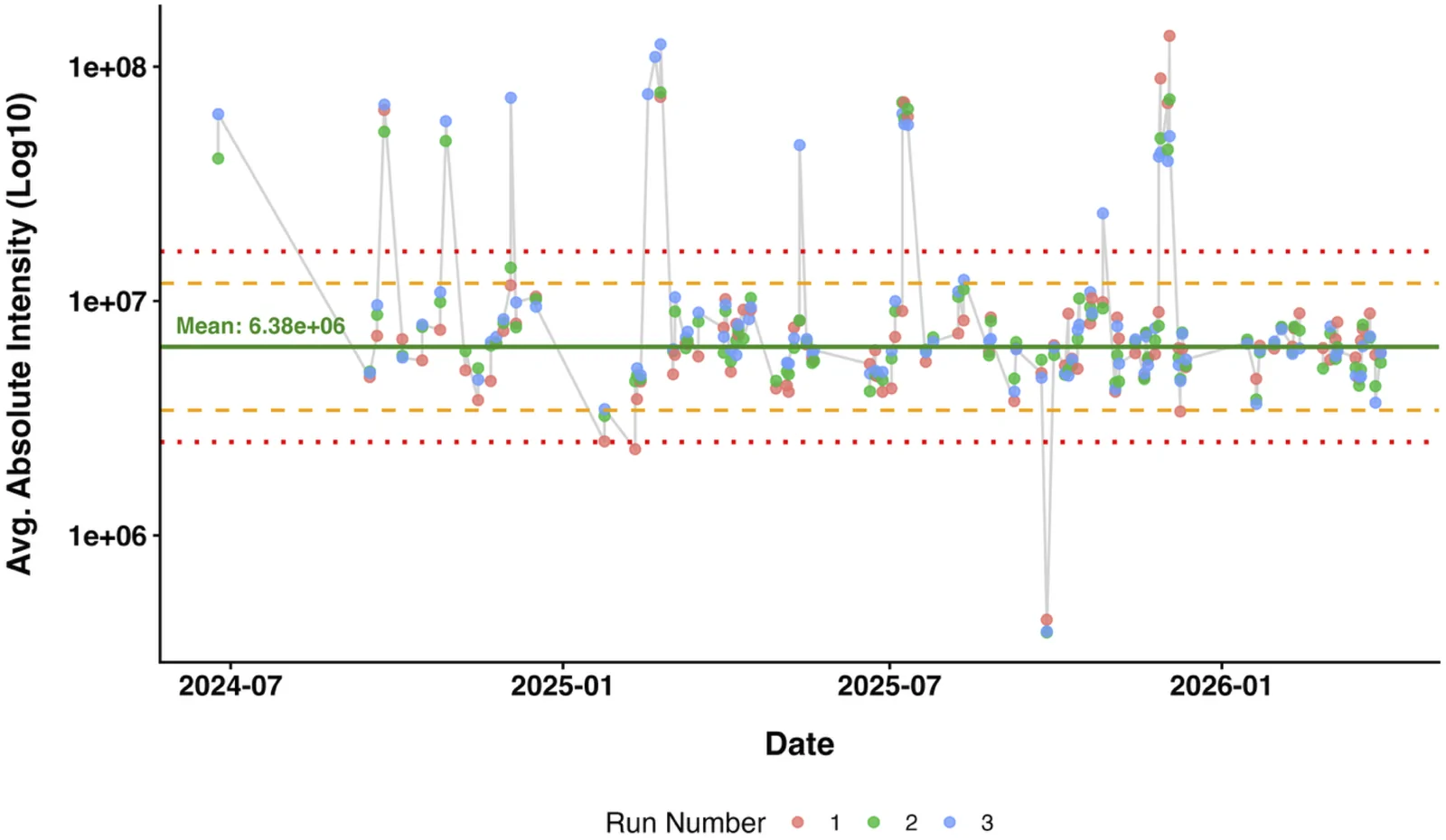
Logarithmic Levey–Jennings QC chart for the positive ion mode D_7_-butyric acid signal over the full study period. The central line represents the geometric mean of the average absolute intensity, while the yellow dashed and red dotted lines indicate ±2 and ±3 standard deviations, respectively. Each point represents one QC measurement and is colour-coded according to the measurement time point within the day. Extreme outliers were excluded from the calculation of the mean and standard deviation by iterative trimming. Measurements outside the ±3 SD range should be flagged for further inspection, potential normalisation, or exclusion depending on the analytical context.

The plot clearly shows that the majority of QC measurements fell within the ±2 SD range, indicating stable and comparable instrument performance for most of the study period. Only isolated measurements or individual measurement days exceeded the ±3 SD threshold. No consistent long-term drift, progressive signal loss, or clear batch effect was observed in the positive ion mode data. Moreover, deviations were not systematically associated with specific maintenance events or with the study duration. These findings indicate that most clinical measurement days were acquired under broadly comparable analytical conditions and that extensive correction or normalisation may not be required for most of the dataset.

The Levey–Jennings chart provides a useful framework for identifying measurement days with abnormal analytical performance. In prospective applications, QC measurements outside predefined control limits could trigger immediate troubleshooting before clinical data acquisition, such as checking the electrospray current, flushing or replacing the capillary, verifying gas flows, cleaning the source, or recalibrating the instrument. In retrospective studies, QC values outside the ±3 SD range can be used to flag clinical measurements for further inspection, sensitivity analysis, mathematical normalisation, or exclusion, depending on the severity of the deviation and the downstream application. Using the ±3 SD threshold has been recommended to detect such “out-of-control” measurements for metabolomics data.^20^ Similar approaches for metabolomics studies using LC-MS or GC-MS, where biological QC samples are used, are common. Features that have a RSD > 20% (LC-MS) or RSD > 30% (GC-MS) are excluded during the QC/QA process.^21^

In clinical breath-analysis studies, complete exclusion of data from days with abnormal measurements may not always be feasible due to recruitment constraints, participant burden, and financial limitations. Therefore, a reference-based QC strategy provides an important decision-support tool rather than a simple pass/fail criterion. When sufficient longitudinal QC data are available, reference-signal behaviour can be used to define expected operational variability, identify analytically concerning deviations, and guide data normalisation strategies. This approach may be particularly valuable for long-term studies, where instrument interventions and maintenance procedures are unavoidable.

### 3.2 Long-term system performance across the clinical study and effect of maintenance operations

The average D_7_-butyric acid signal intensity was calculated for each QC measurement using only spectra recorded between 8 and 10 min, to align to the stable plateau region after equilibration of the reference gas generation system. The average [M + H]^+^ signal intensity of D_7_-butyric acid in positive mode over the entire 21 month monitoring period is shown in Fig. 3 (negative mode in Fig. S3). Gaps in the time series correspond to days where no clinical intervention was scheduled. Overall, the positive-mode D_7_-butyric acid signal showed a RSD of 34% over the 21 month study period. Although individual measurements and selected measurement days showed substantially different signal intensities, no clear long-term drift pattern was observed. Furthermore, signal deviations were not consistently associated with specific maintenance events.

**Fig. 3:**
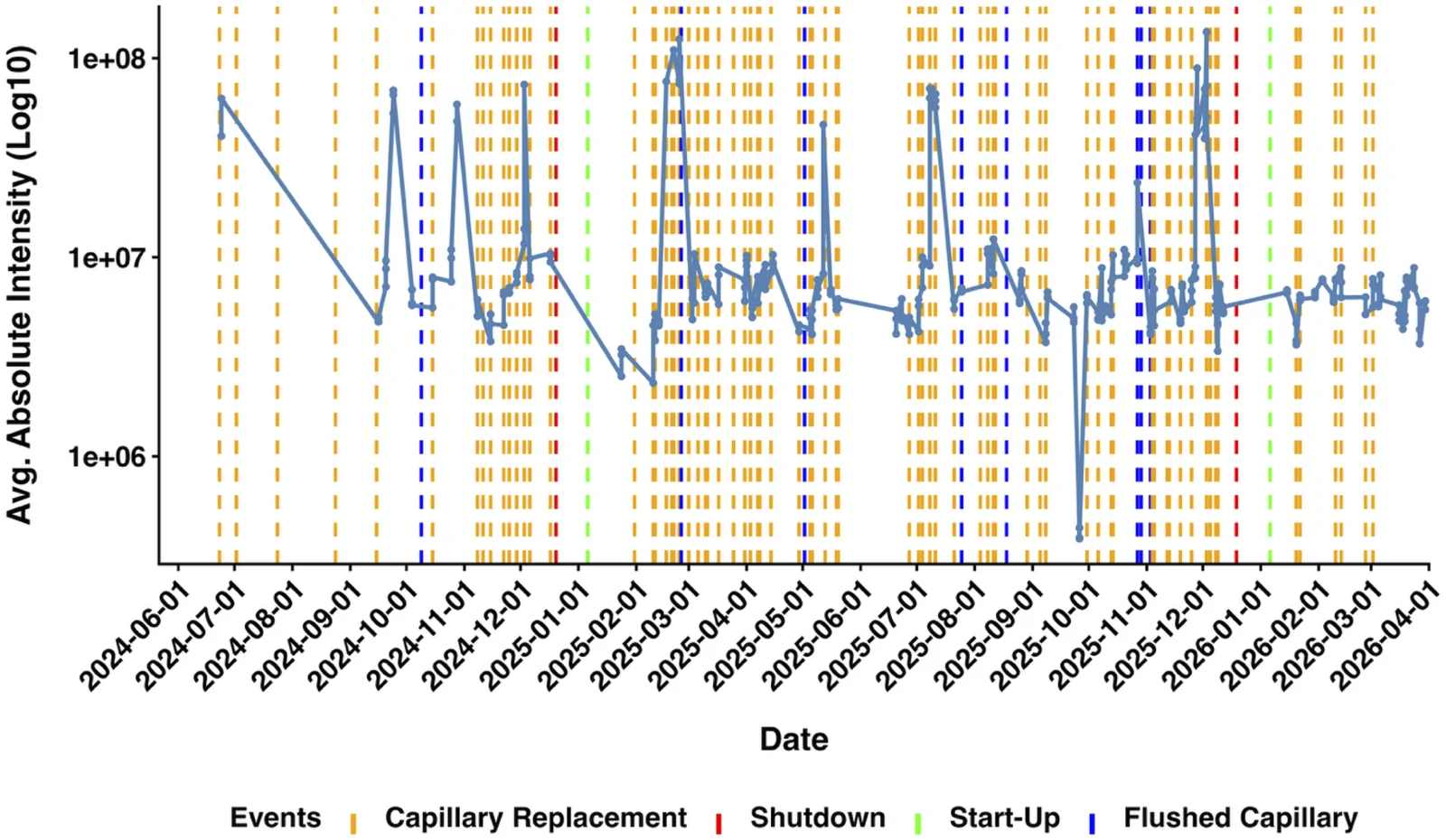
Average absolute intensity on a logarithmic scale of each measurement of the [M + H]^+^ signal of D_7_-butyric acid in positive mode over the whole study period of 21 months. Each point represents one QC measurement. Dashed vertical lines indicate maintenance events, including capillary exchange (*n* = 105), instrument shutdown (*n* = 2), instrument restart (*n* = 2), and capillary flushing (*n* = 8) with a 1 : 1 v/v water/methanol mixture.

Capillary exchanges represented the most frequent intervention events (*n* = 105) and were typically performed when a stable signal could not be obtained. This number represents every physical capillary exchange. However, on some measurement days, multiple capillaries had to be tested before a measurable and stable signal was achieved, resulting in 64 capillaries actually being used for valid measurements. Two longer Orbitrap shutdowns occurred during the holiday periods, followed by instrument restart and more extensive calibration. In both cases, measurements could resume without immediate capillary replacement. After the first shutdown, the first post-startup measurement showed a reduced signal intensity, whereas the second shutdown did not result in a comparable decrease. This suggests that shutdown and restart procedures may affect signal response in individual cases but did not produce a reproducible or systematic effect under the conditions evaluated here.

The lifetime and performance of individual nano-electrospray capillaries were further investigated by comparing the average D_7_-butyric acid signal intensity across the measurement lifetime of each capillary. Substantial differences in capillary lifetime and signal behaviour were observed, as illustrated in signal intensity trajectories for different capillaries in Fig. S2a and b. Several capillaries showed an initial decrease in signal intensity with subsequent stabilization or further degradation, suggesting a possible initial passivation effect followed by progressive emitter degradation, partial clogging, or changes in spray stability. However, the magnitude and timing of this decline varied considerably between individual capillaries. This variability was unexpected, given that the nano-electrospray emitters were commercially available components subjected to manufacturer quality control. In total, 64 capillaries with at least one valid measurement were used, with the number of measurements performed per capillary ranging from 1 to 30 in each mode. The median lifetime of a capillary was 3 measurements per mode, while the arithmetic mean lifespan was 4.8 measurements per mode. This discrepancy of median and mean reflects the observed large variability of capillary lifetimes. Longitudinal analysis of individual capillary lifespans revealed a gradual signal increase (median 0.90% and 6.05% per measurement for negative and positive mode, respectively), supporting the passivation argument. However, capillary exchanges yielded massive signal recoveries, particularly in negative mode (674% *vs.* 57% in positive mode), when comparing the new capillary to the final measurement of the preceding one. Together, these metrics indicate that capillaries maintain stable performance profiles until an abrupt failure necessitates replacement. In the few cases (*n* = 8) where the capillary was flushed to obtain a signal instead of exchanging it, negative mode recovered well (206%) while the positive mode signal dropped by 26% compared to the last valid measurement with the same capillary.

Several factors may have contributed to the observed variation in capillary lifetime. These include crystallisation or residue formation from the electrospray solution, accumulation of particles or dust in the ionisation region, differences in humidity introduced by breath samples, changes in gas-flow stability, or small variations in nitrogen supply and mass-flow control. While such factors may influence the rate at which capillaries degrade or become blocked, they are difficult to control completely in a long-term clinical study setting. Additionally, 42 of the 106 capillaries installed were “dead-on-arrival”, *i.e.*, did not produce a stable electrospray after installation and were discarded without contributing any measurement. This was unexpected and further shows the large variation in capillary performance and quality. Our observations demonstrate that nano-electrospray capillary performance represents one of the main practical challenges for long-term SESI-HRMS operation and should be systematically documented as part of routine QC and maintenance records.

Longitudinal mass accuracy during the study period for the system was also evaluated. The mass error was calculated using the theoretical mass of the D_7_-butyric acid [M + H]^+^ and [M − H]^−^ ions, respectively, and comparing them to the peak with the highest intensity within the ±7.5 ppm mass tolerance window used to generate the XICs. Fig. 4a and b show the mass error for these two ions during the study. With the exception of a few isolated measurements, the mass error was below 5 ppm in the observed time period, staying within an acceptable range.

**Fig. 4:**
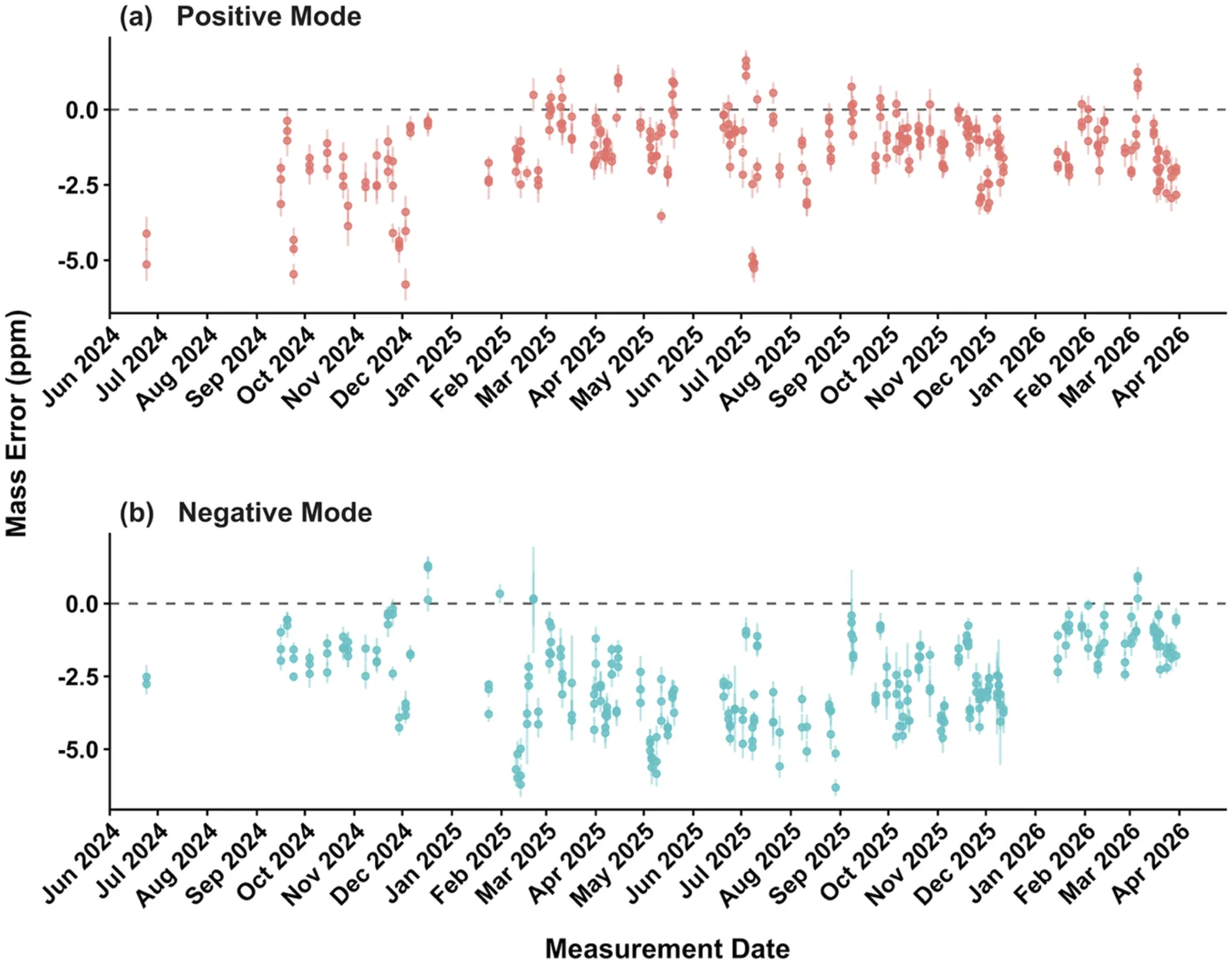
Mass error of the [M + H]^+^ and [M − H]^−^ ions for D_7_-butyric acid in (a) positive and (b) negative mode, respectively. For most of the study duration, the mass error between the detected mass and the theoretical mass was below 5 ppm. Each point represents one QC measurement. The dashed line marks 0 ppm.

These findings indicate that the SESI-HRMS platform maintained broadly comparable performance in positive ion mode across the clinical study, whereas negative-mode performance varied considerably between days (Section 3.3). Routine QC measurements were therefore essential for identifying periods of abnormal analytical behaviour, in particular for negative-mode data, which may require QC-based normalisation before downstream analysis.

### 3.3 Intra-day and inter-day performance across the clinical study

To assess the intra-day performance of the SESI-HRMS and to see if there was an instrumental drift over one measurement day, the average D_7_-butyric acid signal intensities of the three timepoints (morning, mid-day and afternoon) were compared. In both positive and negative ion mode, the overall differences between the three daily measurements were limited, indicating that the instrument did not show substantial drift within a typical clinical intervention day. This is particularly important for breath-analysis studies investigating dynamic metabolic responses, such as postprandial kinetics or pharmacodynamic profiles, where systematic instrumental drift during the measurement day could confound time-dependent biological changes.

The average intra-day relative standard deviation (RSD) for positive ion mode showed lower variability than negative mode (11.6% *vs.* 20.7%), while average inter-day variability for both ionisation modes was considerably higher (30.3% and 81.6% for positive and negative ion mode, respectively). The greater variation in negative ion mode is also evident in Fig. 5 that shows the distribution of daily RSDs by ion mode. The higher variability observed in negative ion mode may reflect reduced electrospray robustness, lower ionisation efficiency, or greater sensitivity to subtle changes in source conditions compared with positive ion mode. Since the same gas generation system, flow parameters, and instrumental platform were used for both polarities, these differences are most likely related to polarity-dependent ionisation behaviour and electrospray stability. Previous studies have shown that ionisation processes in SESI can differ substantially between positive and negative ion modes, and such differences may contribute to the larger intra- and inter-day variability observed for the negative-mode D_7_-butyric acid signal.^22,23^ The relatively large RSD in positive mode could be due to the aforementioned heterogeneous behaviour of the used nanoESI emitters and their frequent exchange, essentially causing the experimental conditions to be different on each measurement day. This could lead to differences in ionisation behaviour also in positive mode and provide a possible explanation for the inter-day variability. Depending on the application, this could possibly be corrected for by adding the capillary as a factor to statistical models that are used in the analysis of the obtained data.

**Fig. 5:**
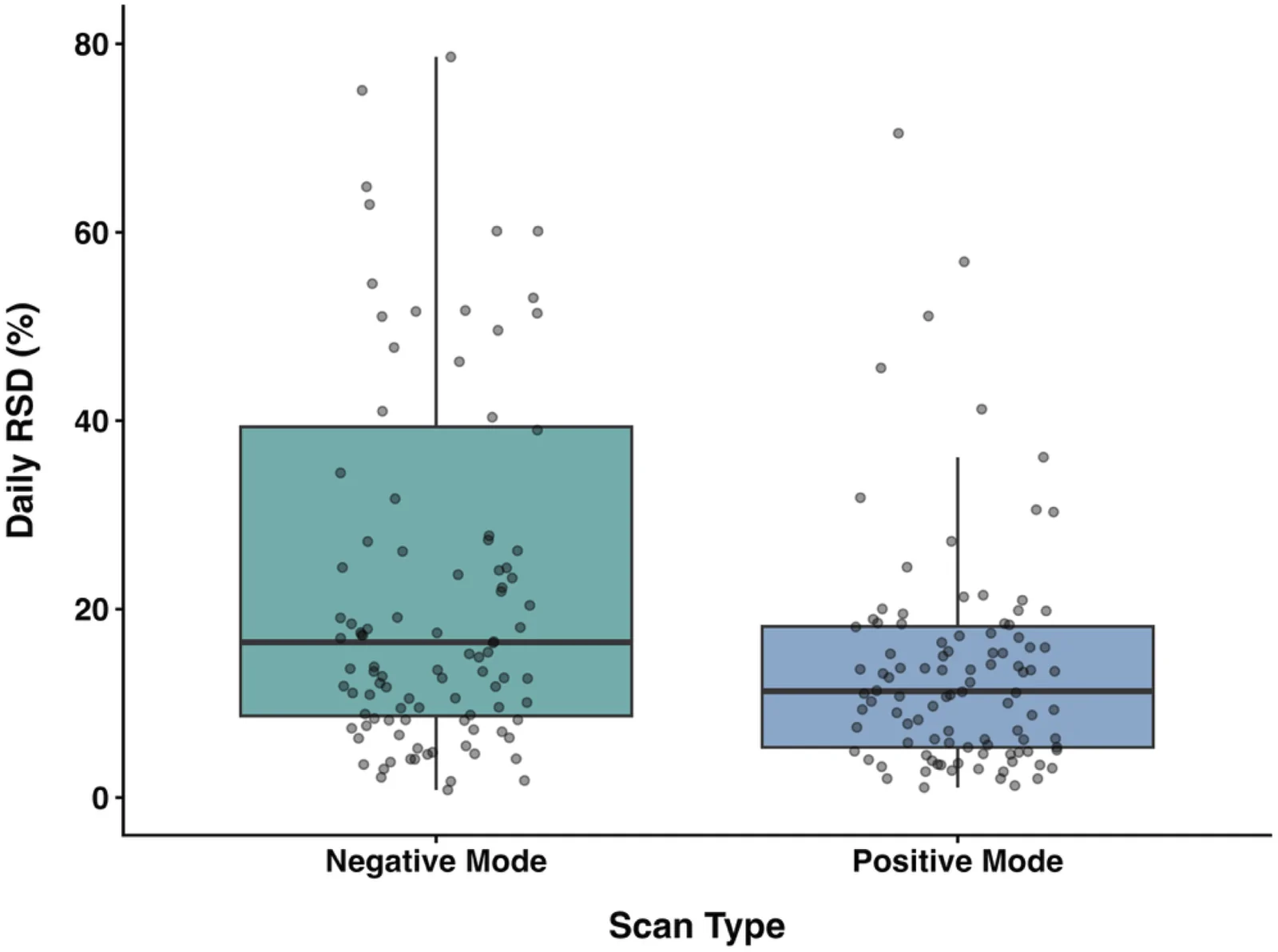
Boxplots of the daily RSD over the whole study period for positive and negative ion mode. Negative ion mode showed a greater variability than positive mode. *n* = 112 assessment days.

Several observations indicate that the high negative-mode inter-day variability arises mainly from the electrospray emitter rather than from the reference gas supply. Positive- and negative-mode acquisitions were recorded directly after each other from the same reference gas, so variations in the delivered concentration would affect both polarities similarly, and the positive-mode inter-day RSD (30.3%) sets an upper limit for this contribution. In contrast, the variance in negative mode arose mainly between days (intra-day RSD 20.7% *vs.* inter-day RSD 81.6%), capillary exchange restored the negative-mode signal far more than the positive-mode signal (674% *vs.* 57%), and signal losses in negative mode coincided with drops in electrospray current (Section 3.4). Negative-mode electrospray of purely aqueous solutions is known to be susceptible to electrical discharge,^22^ and the acidic, aqueous spray solution, chosen for compatibility with the clinical workflow, was not optimised for negative-mode operation. Although D_7_-butyric acid is expected to ionise efficiently as a carboxylic acid, these emitter-related effects are therefore likely to dominate the observed negative-mode variability. Optimising the spray solution for negative mode may reduce this variability in future studies.

Our reported values are higher than in the short-term study of Singh *et al.*, who report an intra-individual RSD of 6.7% over one month for selected breath compounds.^10^ The experimental parameters used differ from ours: different electrolyte for the electrospray (ammonium formate), higher SuperSESI pressure (1.3 bar) and a different supplier for the emitters. This is also reflected in their reported reading of their amperemeter, which showed 130 nA, while we operated in the 160–180 nA range. This in addition to the much shorter observation time could explain why our signal shows higher variability.

On each clinical measurement day, the potential influence of measurement time on the observed D_7_-butyric acid signal was investigated using LMM. The model was applied to log10-transformed intensity data, with measurement day treated as a repeated-measurement structure to account for within-day dependency.

In negative ion mode, measurement time had a statistically significant effect on signal intensity (*P* = 0.005, *F* = 5.54). The first measurement of the day was significantly higher than the second and third measurements (*P* < 0.05), while the second and third measurements were not significantly different from each other (*P* = 0.99). This pattern may indicate an initial settling or ageing effect of the electrospray capillary or ion source, after which the system stabilised during subsequent measurements. Alternatively, it may reflect polarity-specific changes in ionisation efficiency during the first hs of operation.

In positive ion mode, an effect of measurement time was also significant (*P* = 0.007, *F* = 5.10), but a different trend was observed. While the second measurement did not show a significant difference from the first (*P* = 0.78), the third measurement of the day showed a modest but statistically significant increase compared with the first measurement (*P* = 0.007), corresponding to an approximately 11% higher signal.

Several mechanisms may contribute to these patterns. In positive ion mode, the gradual increase over the day is consistent with the progressive signal increase observed over the lifetime of individual capillaries (Section 3.2), which suggests a conditioning or passivation of the emitter during operation. In addition, volatile short-chain fatty acids like butyric acid readily adsorb onto the inner surfaces of tubing and of the ionisation chamber. Repeated exposure to butyric acid during the day may progressively saturate these adsorption sites, so that a larger fraction of the generated reference gas reaches the ionisation region in later measurements. In line with this, the positive-mode signal in the continuous 3 h experiment increased gradually over the first approximately 45 min (Fig. S4a). In negative ion mode, the elevated first measurement coincides with the time of day at which capillaries were typically replaced, corresponding to the observation that capillary exchanges produced much larger signal recoveries in negative than in positive mode (674% *vs.* 57%, Section 3.2). The subsequent decrease may therefore reflect a rapid initial ageing of a freshly installed emitter in negative mode, for example through the onset of electrical discharge. As the continuous experiments were performed outside the clinical study, these results are indicative only, and dedicated experiments would be required to confirm the hypotheses for the observed patterns.

The polarity-dependent direction of the intra-day fluctuations of the butyric acid signal makes it difficult to assign the observed time-of-day patterns to a single mechanism. A plausible explanation is that changes in reference gas concentration, electrospray behaviour, and polarity-specific ionisation efficiency act simultaneously, but influence positive and negative ion mode differently. Since the mechanistic details of SESI ionisation and gas-phase reaction kinetics remain incompletely understood, further dedicated experiments would be required to disentangle these effects. Nevertheless, the observed time-of-day effects were relatively small in positive ion mode and should be interpreted in the context of the broader long-term variability of the platform. Furthermore, the frequency of three measurements per day was established as a compromise for clinical implementation, particularly into our specific study protocol. While continuous or more frequent measurements would most likely capture more intra-day fluctuations, which are present in our data, the three-point assessment provided sufficient resolution to capture relevant signal drift. This sampling timeframe is further justified by the observed stability of our system during a continuous 3 h measurement, ensuring that major deviations are caught without disrupting the clinical workflow.

### 3.4 Stability of the reference gas generation system

The results of the short-term stability (3 h) of the reference gas generation system show that the XIC of the D_7_-butyric acid signal in positive and negative ion mode using the full-scan data (Fig. S4a and b, respectively) rapidly attains a stable signal. Furthermore, the signal drops quickly once the flow to the evaporation chamber is stopped, indicating that the gas generation system has a short response time and no extended washout period is necessary. In positive ion mode, the signal reached a stable plateau within a few minutes and remained relatively constant over the 3 h measurement period. Some fluctuations were observed, which may have resulted from unintentional repositioning or mechanical perturbation of the reference gas system during the experiment but, the overall signal profile indicates that the gas generation system, when evaluated independently of the clinical setup, was able to provide a stable supply of the reference compound over an extended measurement period. A 24 h measurement in positive mode performed before the start of the study (Fig. S5) confirmed that the generator can supply the reference compound over extended periods, but also showed that mechanical perturbation of the evaporation chamber and depletion of the reference solution affect the delivered signal. In negative ion mode, the signal appeared globally more stable over the 3 h acquisition, although one extended signal gap was observed after approximately 25 min, followed by periodic signal drops lasting several scans. During these events, a temporary loss of electrospray current was observed on the ion-source amperemeter, indicating abnormal electrospray behaviour rather than instability of the reference gas generation system. The periodic nature of the subsequent signal drops suggests transient discharge events or intermittent capillary instability, although the exact mechanism cannot be determined from the present data alone. After removing the starting period with the sudden signal drop, the IQR-filtered (to remove the periodic signal losses and outliers) RSD was 18% in positive mode and 8% in negative mode. The higher RSD in positive mode is most likely due to the step increase around 70 min that was probably caused by unintentional repositioning of the evaporation chamber system. Calculating the RSD for the two plateaus separately, 11% and 15% were observed. Singh *et al.*^10^ report a RSD of 2.3% for using β-pinene as a standard for a continuous measurement of one hour. The main differences in our setup are a longer experiment time, higher total flow rate and lower sample flow rate (10 ml min^−1^ sample diluted with 8 L min^−1^*vs.* 1 L min^−1^ sample diluted with 0.5 L min^−1^) and the use of humidified air. These factors can strongly influence the ionisation and spray stability and is probably the cause for our higher RSD. However, since we use similar experimental conditions to a real exhalation (flow rate and humidity), our setup is more likely to reflect the technical variability under real sampling conditions, and we deem the observed variation within an acceptable range. It is important to note that the 3 h continuous experiment was performed after the completion of the clinical study as a standalone characterization of the gas generator. Consequently, it does not reflect the exact state of the system during the clinical study and is not intended to directly validate the longitudinal QC measurements. However, the separate 24 h continuous measurement conducted in positive ion mode prior to the commencement of the clinical study exhibited a similar stability profile. Overall, these findings indicate that the reference gas generation system provides a sufficiently stable and reproducible gas-phase supply of D_7_-butyric acid for routine SESI-MS QC measurements.

Overall, the use of a reference gas generation system enabled objective monitoring of SESI-MS performance over a clinically relevant timeframe. The approach provided information on intra-day stability, inter-day variability, polarity-dependent behaviour, capillary performance, and the impact of maintenance events. These results support the use of reference gas measurements as a practical QC strategy to improve the robustness and interpretability of long-term SESI-MS breath metabolomics studies. We acknowledge however, that one single compound cannot be representative of how the complex mixture of metabolites present in breath might behave under changing instrument performance.^24^ It is very likely, that different compound classes can show drastically different ionisation behaviour that could also differ between ionisation modes, as was seen in our study as well for D_7_-butyric acid.^25^ Implementing a broader range of standards could improve mathematical corrections of the data by enabling compound class-specific performance references. This could be achieved in two ways. First, several evaporation chambers, each loaded with one selected reference compound, could be operated in parallel and merged into the same humidified carrier flow. This allows the concentration of each compound to be adjusted independently *via* its flow rate, at the cost of additional flow controllers. Second, a single evaporation chamber could be loaded with a solution containing a mixture of selected compounds. This leaves the hardware and flow program unchanged, but because compounds with different vapour pressures evaporate at different rates, the composition of the generated gas may change as the solution is depleted during a measurement day, which would need to be characterised beforehand. In both cases, isotopically labelled compounds from different chemical classes, covering the relevant *m*/*z* range and both ionisation modes, would allow class- and polarity-specific performance to be monitored without extending the QC measurement time. In this study, we still opted for a single compound approach to minimize system complexity. A potential future strategy could be to complement the single compound technical variation assessment with a biological QC sample to monitor more metabolite-specific variations, however producing such a sample for on-line breath analysis remains a significant challenge.

## 4 Conclusions

Long-term SESI-HRMS studies require robust quality control strategies to ensure that data acquired over extended periods remain comparable and analytically reliable. This is particularly important for clinical breath-analysis studies, where biological variability, longitudinal sampling, and unavoidable instrument maintenance can complicate data interpretation.

In this study, a custom-built reference gas generation system using D_7_-butyric acid was implemented as a routine QC strategy over 112 clinical measurement days across a 21 month period. In a standalone characterisation, the system provided a stable reference signal over several hours, and the routine QC measurements enabled systematic assessment of short-term, intra-day, inter-day, and long-term SESI-HRMS performance. Positive ion mode showed comparatively stable performance over the study period, whereas negative ion mode displayed considerably greater between-day variability, most likely related to the behaviour of the electrospray emitter, highlighting the importance of polarity-specific QC evaluation.

The repeated reference gas measurements also enabled the identification of abnormal measurement days, assessment of capillary-related variability, and retrospective evaluation of analytical changes in relation to documented maintenance events. Although no major systematic long-term drift was observed, isolated signal deviations were detected, demonstrating the value of routine QC measurements for flagging potentially problematic data. Levey–Jennings-based monitoring provided a practical framework for defining expected operational variability and identifying measurements requiring further inspection, normalisation, or exclusion.

Overall, the reference gas QC strategy we adopted represents a practical and informative approach for monitoring SESI-MS performance in long-term clinical breath-analysis studies. Its implementation can support data comparability, improve confidence in biomarker discovery workflows, and facilitate the broader translation of SESI-HRMS toward robust clinical and longitudinal metabolomics applications.

## Author contributions

AV performed the experiments and analysed the data. AV, RZ, KJB, DP, and SG interpreted the compiled results. AV and SG prepared the manuscript. All authors contributed to the discussion and revisions of the paper.

## Conflicts of interest

There are no conflicts to declare.

## Supplementary Material

AY-OLF-D6AY01843B-s001

## Data Availability

The original data used in this publication are made available in a curated data archive at ETH Zürich (https://www.research-collection.ethz.ch) under the http://hdl.handle.net/20.500.11850/805680. Supplementary information (SI) is available. See DOI: https://doi.org/10.1039/d6ay01843b..
