# Supplementary material for: Long-term performance monitoring of a secondary electrospray ionisation high-resolution mass spectrometry platform using a vapor generator for routine quality control
Source: Anal Methods. 2026 Oct 9. Online ahead of print. doi: 10.1039/d6ay01843b (PMC13662781; doi:10.1039/d6ay01843b)
Supplement: Supplementary file 1 [file AY-OLF-D6AY01843B-s001.pdf]

Supplementary Information (SI) for Analytical Methods.
This journal is © The Royal Society of Chemistry

Supporting Information

Long-term performance monitoring of a secondary electrospray ionisation high-resolution mass spectrometry platform using a vapor generator for routine quality control

Albin Vadakkechira[1], Renato Zenobi[1], Daniel Pohl[2], Kathryn J. Burton-Pimentel[3], Stamatios Giannoukos[1,3,*]

Affiliations:

[1] Department of Chemistry and Applied Biosciences, ETH Zürich, Zurich, Switzerland

[2] Department of Gastroenterology and Hepatology, USZ, Zurich, Switzerland

[3] Agroscope, Bern, Switzerland

Address correspondence to: Stamatios Giannoukos, Department of Chemistry and Applied Biosciences, ETH Zürich, Vladimir-Prelog-Weg 3, 8093, Zürich, Switzerland, Tel: +41 44 632 61 12, stamatios.giannoukos@org.chem.ethz.ch

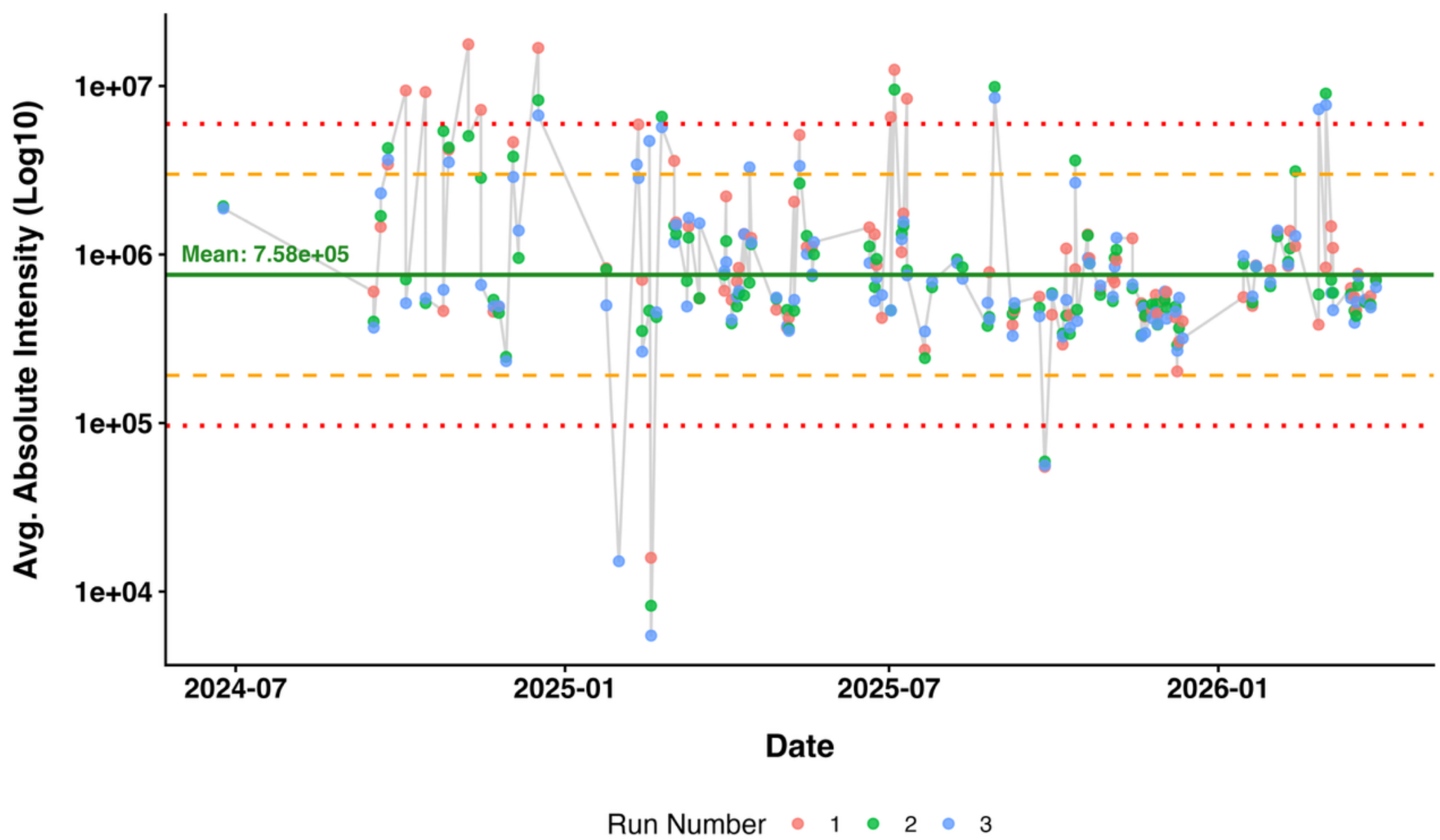

Figure S1. Logarithmic Levey-Jennings QC chart for negative ion mode D_7-butyric acid signal over the full study period. The central line represents the geometric mean of the average intensity, while the dashed and dotted lines indicate ±2 and ±3 standard deviations, respectively. Each point represents one QC measurement and is colour-coded according to the measurement time point within the day.

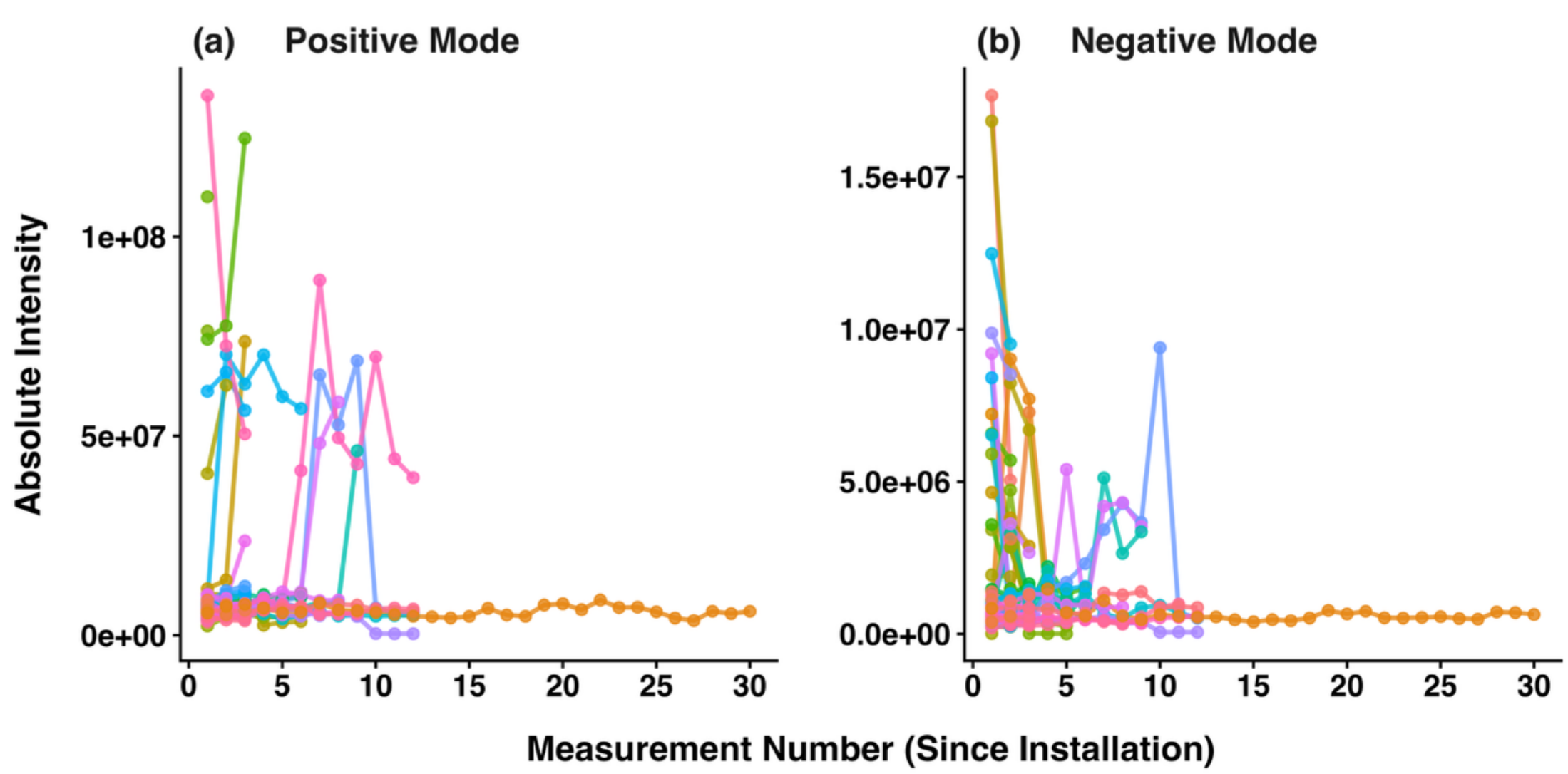

Figure S2. Comparison of the average D_7-butyric acid signal intensity across the lifetime of individual electrospray capillaries in positive (a) and negative (b) ion mode. Each point represents a QC measurement, and capillary lifetime is expressed as the number of measurements performed since the respective capillary's installation.

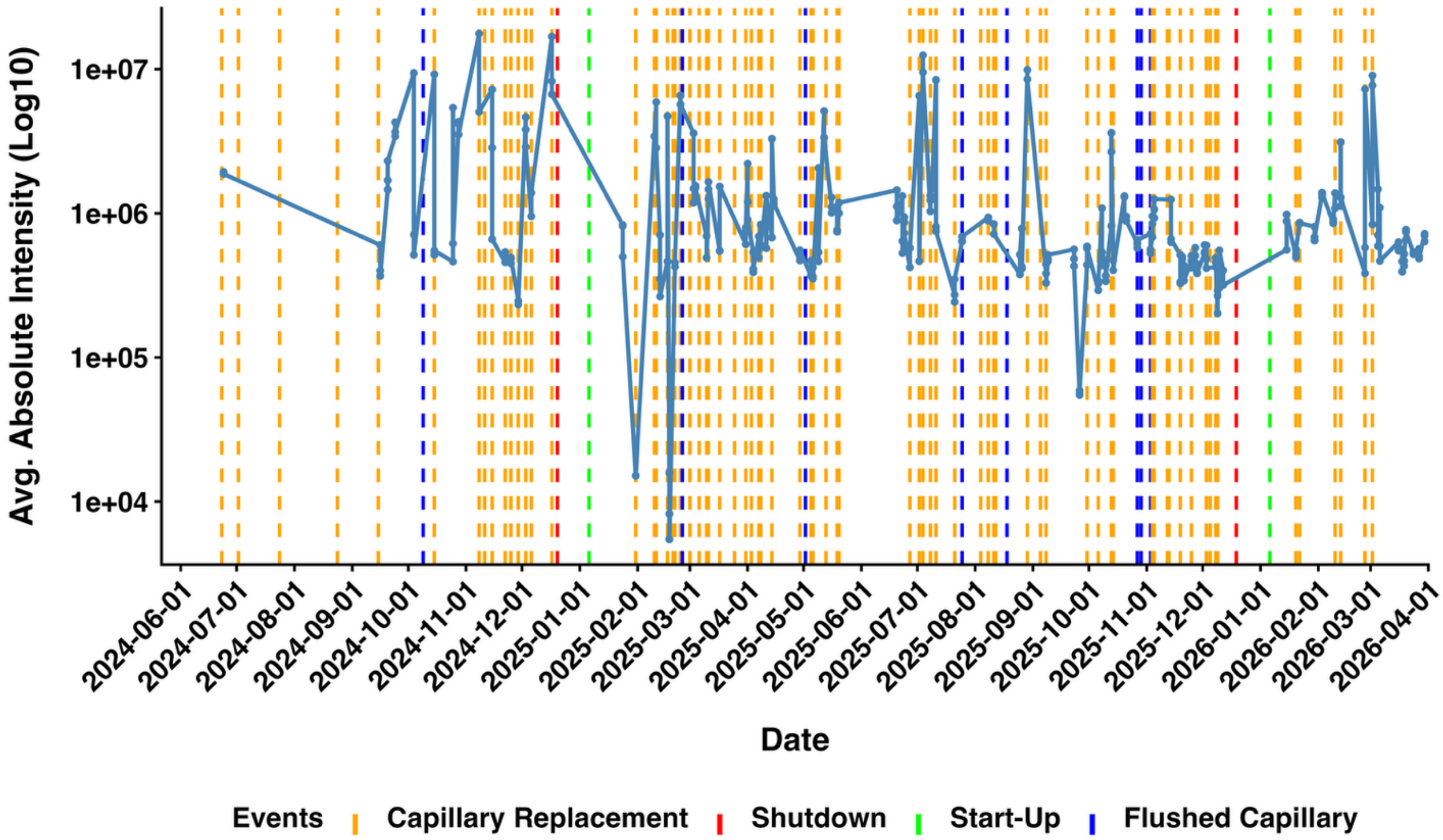

Figure S3. Average absolute intensity on a logarithmic scale of each measurement of the [M-H]- signal of D7-butyric acid in negative mode over the whole study period of 21 months.

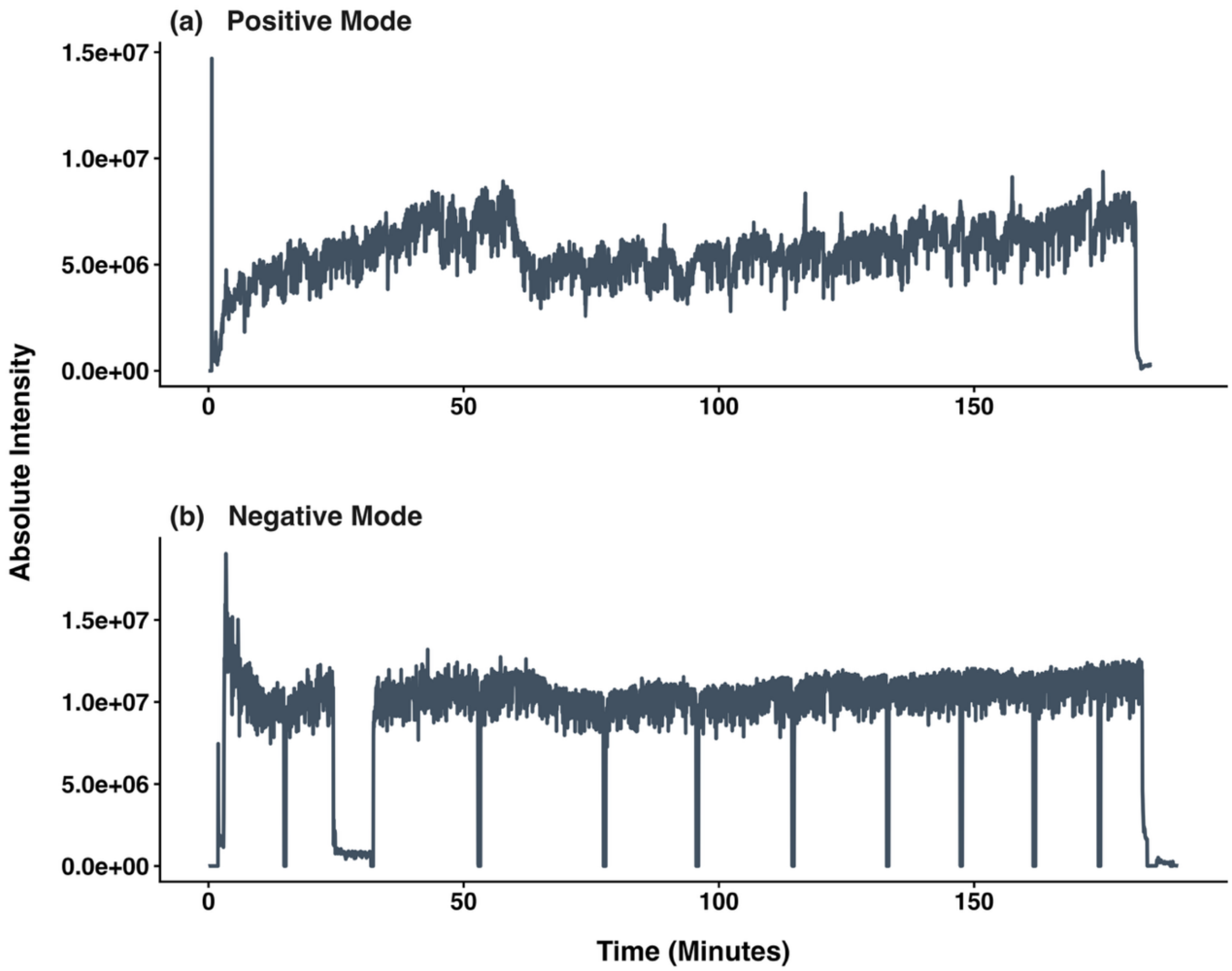

Figure S4. (a) XIC of the raw $[M + H]^+$ signal of D_7-butyric acid in positive mode over the period of 3h using intensities from the full scans only. (b) XIC of the raw $[M - H]^-$ signal of D_7-butyric acid in negative mode over the period of 3h using intensities from the full scans only.

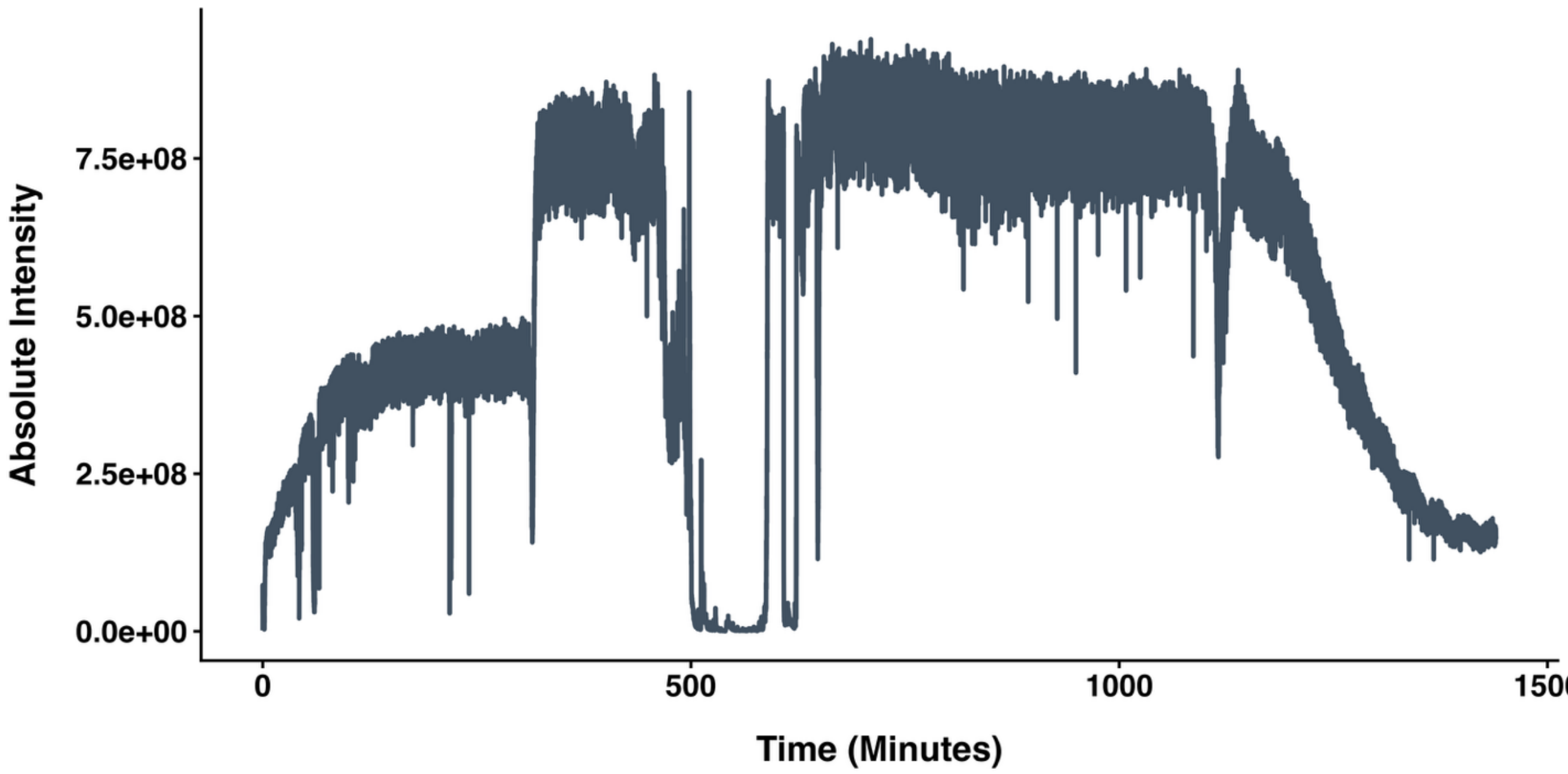

Figure S5: XIC of the raw [M + H]+ signal of D7-butyric acid in positive mode over the period of 24h using intensities from the full scans only. The observed signal drop after 500 min was associated with a total loss of signal due to unknown reasons. The step increase of the signal plateau is likely due to repositioning of the evaporation system. The gradual decline towards the end of the measurement is likely due to depletion of the solution in the evaporation chamber.
